# Associations between self-reported depression and functioning across ICF domains: a Danish population-based study

**DOI:** 10.3389/fresc.2026.1793735

**Published:** 2026-05-18

**Authors:** Pernille Pedersen, Merjema Zulfikari, Marc Sampedro Pilegaard, Dorte Balle Rubak, Charlotte Ibsen

**Affiliations:** 1 DEFACTUM, Central Denmark Region, Aarhus, Denmark; 2 Department of Public Health, Aarhus University, Aarhus, Denmark; 3 Department of Social Medicine and Rehabilitation, Gødstrup Hospital, Gødstrup, Denmark; 4 Department of Clinical Medicine, Aarhus University, Aarhus, Denmark

**Keywords:** adults, depression, disability, functioning, health survey, International Classification of Functioning, Disability and Health (ICF)

## Abstract

**Background:**

Depression is a major contributor to disability, yet clinical assessment and research have primarily focused on symptom severity. This study aims to examine associations between self-reported depression and functioning across the International Classification of Functioning, Disability and Health (ICF), while accounting for environmental and personal factors.

**Methods:**

The study analyzed self-reported data from the 2021 Danish “How are you?” survey. Guided by the ICF framework, associations between self-reported depression and functioning were analyzed using logistic regression in a stratified random sample of 48,936 adults aged 25 years and older.

**Results:**

Of the 28,101 respondents included, 3,060 (13%) reported symptoms indicative of depression. Across all ICF components, individuals with self-reported depression reported substantially more disability than those without depression. After adjustment for personal and environmental factors, depression (reference: no depression) remained strongly associated with impairments in body functions and structures, including poor sleep quality (OR 2.94, 95% CI 2.44–3.54), atypical sleep duration (OR 1.27, 95% CI 1.18–1.37), perceived stress (OR 9.75, 95% CI 8.12–11.72), life strain (OR 1.97, 95% CI 1.67–2.33), and loneliness (OR 2.93, 95% CI 2.43–3.52). Depression was also associated with activity limitations and participation restrictions, including requiring assistance with activities of daily living (OR 1.39, 95% CI 1.10–1.74), difficulty engaging with healthcare providers (OR 1.83, 95% CI 1.44–2.32), non-engagement in work or education (OR 1.40, 95% CI 1.10–1.79), and lack of community involvement (1.19, 95% CI 1.03–1.39).

**Conclusions:**

Self-reported depression was associated with widespread disability across psychological, social, and physical domains, underscoring its relevance as a condition with substantial disability. The findings support the utility of ICF for systematically capturing the multidimensional impact of depression at the population level, informing comprehensive assessment and rehabilitation-practice and research.

## 1 Introduction

Depression is among the most prevalent mental disorders worldwide, affecting an estimated 280 million individuals (1). It is characterized by persistent low mood, reduced energy, and diminished interest, often accompanied by impaired concentration, sleep disturbances, appetite changes, and feelings of worthlessness or guilt (2). Beyond these clinical symptoms, depression is a major contributor to years lived with disability globally (1) and is strongly associated with disability across multiple domains, including cognition, mobility, self-care, employment, social relationships, household activities, and community participation (3–8). These limitations reduce quality of life and hinder return to work. There is a bidirectional association between depression and disability, whereby each can increase the risk of the other (9, 10).

A clear distinction between symptoms and functioning is essential, as several diagnostic criteria, such as insomnia and concentration problems, overlap with functioning dimensions, but do not capture the full extent of participation restrictions. Broader consequences, including unemployment, absenteeism, and social difficulties, reflect how depression disrupts everyday life. Capturing the lived experience of depression, therefore, requires attention to contextual factors (4, 5). Nevertheless, most clinical assessments and research studies prioritize symptom severity, while giving limited attention to outcomes that reflect everyday life such as work participation, social relationships, and daily activities. Evidence shows that disability frequently persists even when symptoms have remitted, underscoring that depression affects multiple domains of functioning and cannot be understood through symptom severity alone (11). Assessing outcomes that capture everyday capabilities therefor provides crucial insight into the practical and social consequences of depression and support a biopsychosocial approach to clinical assessment and treatment (12).

The World Health Organization's International Classification of Functioning, Disability, and Health (ICF) offers a biopsychosocial framework well suited to understanding depression-related disability (13). ICF conceptualizes functioning and disability as a dynamic interaction between a person's health condition and contextual factors (environmental and personal), encompassing the components of body functions, body structures, activities and participation (13). Building on this framework, the ICF Core Set for Depression was developed to capture the most relevant aspects of functioning and disability in individuals with depression (14). The Core Set serves as a practical tool for assessment, research, and rehabilitation planning, providing a comprehensive understanding of how depression affects everyday life (14).

Despite substantial evidence linking depression to disability, most studies examine isolated domains, such as cognitive functioning or work participation, thereby limiting the ability to understand functioning in a more holistic way. Moreover, existing research has predominantly focused on individuals with major depressive disorder (3–5, 8, 12, 15), despite evidence that mild and moderate symptoms are also associated with disability (7, 16) and are linked to an increased risk of progression to more severe depressive disorders (17). A broader biopsychosocial perspective is therefore needed, one that captures the full range of symptom severity and considers functioning across multiple domains (4). Such knowledge is particularly relevant in primary care, where early assessment of disability may help guide timely interventions and reduce the risk of persistent long-term disability (12). Thus, this Danish population-based study aimed to examine associations between self-reported depression and functioning, as conceptualized by the ICF components of body functions, body structures, activities and participation, while accounting for relevant environmental and personal factors.

## 2 Materials and methods

### 2.1 Study design and setting

This cross-sectional study utilized self-reported data from the 2021 population-based health survey “How are you?”, conducted in the Central Denmark Region (18). In 2021, approximately 23% of the Danish population resided in this region (19).

### 2.2 Study population and data collection

The survey was based on a stratified random sample of 48,936 citizens aged 25 years and older, drawn from the Danish Civil Registration System (CPR-register), who were invited to participate. Data were collected between February and May 2021 (18), using a questionnaire available in both web-based and postal formats. The web-based questionnaire was distributed via the secure digital mailbox linked to each individual's personal identification number. To maximise the response rate, three reminders were sent to recipients of the postal questionnaire, and 3–4 reminders were sent to those invited to complete the web-based version.

### 2.3 Exposure variable

The exposure variable was self-reported depression, assessed using the Major Depression Inventory (MDI), a validated 12-item questionnaire measuring depressive symptoms experienced during the preceding two weeks. The total MDI score ranges from 0 to 50, with higher scores indicating greater symptom severity (20). In accordance with established cut-off values, respondents were categorized as *no depression* (<21 points) and *depression* (≥21 points) (21).

### 2.4 Selection of outcome variables

**Figure 1:**
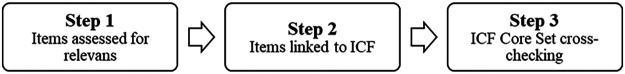
Three-step process for selecting outcome variables. The figure illustrates the sequential steps used to identify and select outcome variables in relation to the International Classification of Functioning, Disability and Health (ICF).

In total, the survey comprises 299 items addressing a broad range of domains, including self-rated health, chronic disease and multimorbidity, mental health (stress, depression, loneliness), health literacy, wellbeing, social relations, lifestyle factors (smoking, alcohol, physical activity), work environment, and utilization of health services. As mentioned above, we first identified items addressing self-reported depression (exposure variable) using the 12-item MDI. This left 287 remaining items to be assessed as potential outcome variables. The selection of outcome variables was conducted in a systematic three-step process by two authors (MZ and CI), during which items were excluded as appropriate (Figure 1).

Step 1: Items were assessed for their relevance to the study aim of describing functioning. A total of 65 items were considered conceptually irrelevant to functioning and therefore excluded. The excluded items concerned COVID-19 (11 items), tattoos (15 items), sexual assaults (7 items), diet (27 items), and free-text responses (5 items).

Step 2: The remaining 222 items were linked to ICF using the refined ICF Linking Rules to identify items describing aspects of functioning (22). Two authors (MZ and CI) independently identified the meaningful concepts within each item. The linking process was organized in Microsoft Excel 2007 using a standardised table to ensure consistency and traceability. Each meaningful concept was linked to the most specific ICF category (e.g., d450 Walking). The two authors subsequently compared and discussed their individual linking results, resolving discrepancies through discussion until consensus was reached. When consensus could not be reached, a third researcher with extensive expertise in ICF classification and linking procedures was consulted to determine the final linking result, thereby ensuring consistency and methodological rigor. Items linked to environmental factors (19 items), as well as those classified as “not defined” (41 items) and “not covered” (3 items) according to ICF liking rules, were excluded. Environmental factors were not considered as outcomes, as the study focused on functioning within body functions and structures, and activities and participation. According to ICF linking rules, items classified as “not defined” or “not covered” were excluded, as they cannot be meaningfully assigned to specific ICF categories (22). This resulted in 159 items linked to relevant ICF categories for analysis.

**Figure 2:**
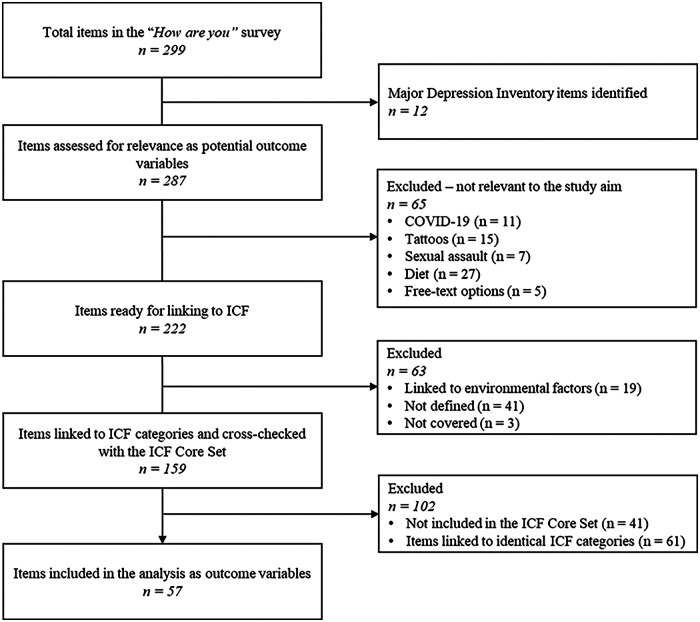
Flow of item selection and ICF linking process for inclusion in the analysis. Flow diagram illustrating the selection of questionnaire items and the International Classification of Functioning, Disability and Health (ICF) linking process for inclusion in the analysis.

Step 3: The linking of the 159 items was subsequently cross-checked with ICF categories included in the Core Set for Depression to identify which items should be retained for analysis (14). The Core Set is available in two versions: The Comprehensive ICF Core Set, which includes ICF categories for all relevant aspects of functioning of depression (121 ICF categories), and the shorter, brief Core Set with ICF categories for the most central aspects (31 ICF categories) (14). Items linked to ICF categories not represented in either of the core sets were excluded (41 items). In cases where multiple items were linked to identical ICF categories, the authors (CI and PP) jointly selected the most conceptually relevant item for the study aim, thus 61 items were excluded. The three-step selection process resulted in 57 items to be included as outcome variables (Figure 2).

Table 1 presents an overview of the outcome variables, followed by a detailed description of each variable.

**Table 1:** Outcome variables, no of items, measures and corresponding ICF categories.

Outcome variables	No of items	Measures	ICF categories (2nd level)
*Body functions and body structures*			
Sleep quality	5 items	Adapted from Pittsburgh Sleep Quality Index	b134 Sleep functions
Sleep duration	1 item		b134 Sleep functions
Perceived stress	10 items	The Perceived Stress Scale	b152 Emotional functions
Pain	4 items	Adapted from the Nordic musculoskeletal questionnaire	b280 Sensation of pain
Life strain	9 items	Adapted from the Life event Questionnaire	b152 Emotional functions
Loneliness	3 items	Three-Items Loneliness Scale	b152 Emotional functions
Body mass index (BMI)	2 items	Calculated as body weight in kilograms divided by height in metres squared (kg/m^2^)	b530 Weight maintenance functions
*Activities and participation*			
Physical activity	1 item	The WHO global guidelines on physical activity	d570 Looking after one's health
Activities of daily living (ADL)	1 item	Translated and nationally adapted version of an original EU-standardised item from the European Health Interview Survey	d230 Carrying out daily routine
			d620 Acquisition of goods and services
			d630 Preparing meals
			d640 Doing housework
			d650 Caring for household objects
Health literacy	10 items	Two sub-scales from the Australian Health Literacy Questionnaire	d166 Reading
			d170 Writing
			d310 Communicating with—receiving—spoken messages
			d330 Speaking
			d350 Conversation
			d570 Looking after one's health
Work- and educational status	2 items	-	d845 Acquiring, keeping and terminating a job
			d830 Higher education
Work ability	1 item	Adapted from Work Ability Index	d850 Remunerative employment
Social contact	7 items	Adapted from Valtorta's social isolation index	d750 Informal social relationships
			d760 Family relationship
Community life	1 item	-	d910 Community Life
			d920 Recreation and leisure

#### 2.4.1 Body functions and body structures

Sleep Quality (b134) was assessed using five items from the Pittsburgh Sleep Quality Index, referring to experiences during the past four weeks (23). The items addressed feeling rested, difficulty falling asleep, night awakenings, early awakenings, and restless sleep. The first item had three response options (“Yes, usually”/“Yes, but not often enough”/“No, never (almost never)”, while the remaining items were rated on a four-point Likert scale (“Not during the past 4 weeks”/“Less than once a week”/“Once or twice a week”/“Three or more times a week”). Responses were converted to a 0–100 scale, with higher scores indicating poor sleep quality. A validated cut-off score of ≥80 indicated poor sleep quality (18, 24). Accordingly, sleep quality was dichotomised into poor (≥80 points) and good (<80 points).

Sleep duration (b134) was assessed using one item from the Pittsburgh Sleep Quality Index (23). Self-reported hours and minutes of nightly sleep were converted into total minutes. The variable was then dichotomised into 7–9 h per night [recommended sleep duration for adults (25)] vs. atypical sleep duration (< 7 or >9 h).

Perceived stress (b152) was assessed using the 10-item Perceived Stress Scale (PSS-10), measuring subjective stress over the past month (26, 27). Total scores range from 0 to 40, with higher scores reflecting greater stress. As no universal cut-off exist, stress was dichotomised as no stress (<18 points) and stress (≥18 points), based on previous research (28).

Pain (b280) was assessed with four items adapted from the Nordic Musculoskeletal Questionnaire: asking: “Have you, within the past 14 days, been bothered by any of the following types of pain and discomfort?” (29). The four pain types included shoulder/neck pain, extremity/joint pain, back pain, and headache. Respondents who reported being severely bothered by pain in at least one of these areas were classified as having pain; all others were classified as having less pain.

Life strain (b152) was assessed by nine items adapted from the Life Event Questionnaire asking: “Within the past 12 months, have you felt burdened by any of the following?” (30). Nine potential sources of strain were listed: (1) personal finances, (2) housing, (3) work, (4) relationship with partner, (5) relationships with family/friends, (6) personal illness, (7) illness within family member or close friend, (8) bereavement, and (9) other strain. Items were rated on a four-point scale (“no”/“yes”/“a little”/“yes, somewhat”/“yes, a lot”). Respondents who answered “yes, somewhat” or “yes, a lot” on at least two items were classified as experiencing life strain, while the rest were classified as not experiencing life strain.

Loneliness (b152) was assessed using the Danish version of the Three-item Loneliness Scale (TILS) (31). The items: “How often do you feel isolated from others?”, “How often do you feel you lack companionship?” and “How often do you feel left out?”, were rated on a 3-point Likert scale (“hardly ever”/“sometimes”/“often”). Items' scores were summed to create a total score ranging from 3 to 9, with higher scores indicating greater loneliness. The variable was dichotomised, with scores of 7–9 classified as lonely.

BMI (b530) was calculated from self-reported height and weight and classified according to WHO criteria: underweight (<18.5), normal weight (18.5–24.9), overweight (25.0–29.9), and obesity (≥30.0) (32). For analysis, respondents were grouped into two categories: underweight/normal weight (<25.0) and overweight/obese (≥25.0).

#### 2.4.2 Activities and participation

Physical activity (d570) was assessed with the question: “How many days per week are you physically active for at least 30 min per day?” in line with the WHO global guideline on physical activity (33). Respondents reporting physical activity on 0–1 days per week were classified as physically inactive.

ADL (d230, d620, d630, d640 and d650) was assessed using one item translated and nationally adapted from an original EU-standardised item from the European Health Interview Survey, asking: “Do you, due to illness or other health problems, need assistance from friends, family, or home care, etc., to manage your daily tasks?” (34). Respondents reporting no need for assistance were categorized as “no need for help”, whereas those indicating a need for assistance were categorized as “need for help”.

Health literacy (d166, d170, d310, d330, d350 and d570) was assessed using 10 items from two subscales from The Health Literacy Questionnaire (35); (1) Understanding health information well enough to know what to do and (2) Actively engaging with healthcare providers. Each subscale includes five items rated on a four-point Likert scale from “very difficult” to “very easy”. Mean scores were calculated and standardised from 1 (lowest) to 4 (highest). Respondents with mean scores ≤2 were categorized as having difficulty understanding health information or difficulty engaging with healthcare providers, whereas scores >2 were categorized as no difficulty understanding health information or no difficulty engaging with healthcare providers (36).

Work and educational status (d845 and d830) were assessed using two items; “Are you currently working?” and “Are you currently enrolled in education?” Responses were categorized as either engaged in work or education or not engaged.

Work ability (d850) was assessed with one item: “Do you feel that your ability to work is reduced?” (37). This question refers to a permanent reduction in work ability rather than a temporary limitation due to illness or similar causes. Respondents answering “yes”/“very much”/“yes, a lot” were classified as having reduced work ability, whereas those answering “yes, a little” or “no” were classified as having no reduced work ability.

Social contacts (d750 and d760) were assessed using seven items adapted from the Valtorta's Social Isolation Index, covering social contacts with family, friends and acquaintances (38). Contacts include face-to-face meetings, phone calls, emails, social media, online games, or video calls. Respondents rated contacts frequency on a Five-point Likert scale from “daily/almost daily” to “never”. Responses of “less than once a month” and “never” were scored as 1, and all others 0, yielding a total score of 0–7, with higher scores indicating reduced social contacts. Scores were categorized as reduced (4–7 points) or good (0–3 points).

Community life (d910 and d920) was assessed with the question: “How often do you participate in community activities or volunteer work with others?”. Participation included involvement in sports clubs, social work, professional associations, board work, neighborhood groups, musical or cultural organizations, or religious gatherings. Respondents participating weekly, monthly, or rarely were classified as involved in community life, while those reporting never participating were classified as not involved in community life.

### 2.5 Selection of covariates

Items from the survey, that were linked to ICF categories under environmental or personal factors, were assessed as potential covariates, as these factors may influence the association between depressive symptoms and functioning. Environmental factors are classified within ICF, whereas personal factors are recognized but not yet classified (39). The selection of environmental factors followed Steps 2 (Linking to ICF) and 3 (cross-checking with the ICF Core Set), with emphasis on including only the most relevant variables. This selection was constrained by the available survey items, which did not comprehensively capture environmental factors as defined within the ICF (e.g., products, technology, or physical environment). Consequently, the included variables should be interpreted as proxies for environmental context, that is, indirect indicators of the individua's surroundings rather than direct measures of environmental barriers or facilitators. For example, living with a partner is not a direct measure of social support, but may indicate aspects of the individual's social context. These variables were included solely as covariates to adjust for potential confounding, and their number was restricted to minimize the risk of overfitting (Figure 1). As personal factors are not classified in ICF, their selection was guided by existing evidence on factors known to influence functioning such as sociodemographic characteristics and lifestyle-related variables (40–42). The final selection was determined through consensus between two authors (PP and CI), based on relevance to the study aim and conceptual alignment with the ICF model.

Environmental Factors included living with a partner (e310), treatment recipient (e355), income (e165) and social security benefits (e570) representing 12 items. These variables were selected because they are included in the ICF Core Set for Depression (14). Living with a partner was categorized as living alone or with others (spouse/partner). Healthcare use was classified as receiving treatment or rehabilitation vs. not. Income was grouped as 0–149,000 DKK, 150,000–399,000 DKK, or ≥400,000 DKK. Social security benefits were dichotomised as receiving (disability pension, unemployment benefits, sickness benefits, cash benefits, or flexible job scheme) vs. not receiving any.

Personal Factor included age, gender, educational attainment level, native language, smoking, and alcohol representing 11 items. These variables were selected as covariate because they are known to influence both the prevalence and reporting of depressive symptoms as well as functioning, and may therefore confound the association between self-reported depression and functioning (40–42). Age was grouped into six categories (25–34, 35–44, 45–54, 55–64, 65–74, and ≥75 years). Gender was classified as male or female. Educational attainment level was categorized as (1) No formal education or basic courses, (2) Short-cycle tertiary education or vocational education, and (3) Medium- or long-cycle tertiary education. Native language was categorized as Danish or another. Smoking status was classified as smoker (daily, weekly, or less than weekly) or non-smoker (quit or never smoked). Alcohol use was defined by the frequency of consuming ≥5 drinks on one occasion and categorized as frequent (daily, weekly, or monthly) or infrequent (rarely or never).

### 2.6 Statistical analysis

Characteristics for outcome variables and covariates are presented using descriptive statistics, reported as the number of respondents and frequency (*n*/%), stratified by depression status. Associations between depression and functioning variables were analyzed using unadjusted and adjusted logistic regression. The analyses were designed to examine associations rather than causal pathways. Accordingly, the analyses focused on estimating overall associations, and potential effect modification was not explored; therefore, no interaction terms were included.

Missing data were handled using a pragmatic approach to minimise information loss while maintaining data quality. Excluding respondents with incomplete data may reduce statistical power and introduce bias if data are not missing completely at random (43). For multi-item scales (MDI, perceived stress, and loneliness), missing values were imputed using the individual's mean score when a limited number of items were missing (≤3 for MDI, ≤2 for perceived stress, and ≤1 for loneliness). For MDI, respondents exceeding this threshold were excluded from the study sample. For other measures, scale-specific criteria were applied [complete-case requirements for sleep quality, exclusion if ≥3 items were missing for life strain, ≥2 items were missing for social contacts, and for health literacy (36)]. For composite measures based on multiple items, such as pain those with missing responses were excluded from the respective analyses. For single-item variables or measures based on few items (sleep duration, BMI, ADL, work and educational status, and community life), respondents with missing data were likewise excluded. Overall, missing outcome data was handled at a variable level. Respondents were excluded only from the specific analyses in which the variable was included, resulting in varying sample sizes across analyses.

In Model 1, estimates were adjusted for other functioning variables related to body functions and structure, as well as activity and participation. Model 2 was further adjusted for environmental and personal factors. As the adjusted models may include variables on the pathway between depression and functioning, estimates should be interpreted as associations rather than causal effects. As several functioning variables may be correlated, multicollinearity cannot be excluded and may have influenced the stability of some estimates.

Due to variations in response rates, the analytic sample size differed across outcome variables. All estimates are reported as odds ratios (OR) with 95% confidence intervals (CI). A *p*-value <0.05 was considered statistically significant. Calibrated sampling weights were applied to enhance the representativeness of the study population these weights accounted for survey design and non-response using a model-based calibration approach developed by Statistics Denmark, incorporating factors such as sex, age, municipality, social background, and healthcare utilization (44, 45). All analyses were conducted using Stata/MP version 19.5.

### 2.7 Ethics

Data collection and use of data for the purpose of this study were approved by the Danish Data Protection Authority (ref. 2012-58-0006) and the Central Denmark Region (ref. 1-16-02-571-13) and conducted in accordance with the Helsinki Declaration. In accordance with Danish legislation, studies based solely on questionnaire and/or registry data do not require approval from the Committee on Health Research Ethics (§14, subsection 2). All respondents received written and online information about the study and its purpose. Informed consent was obtained through voluntary completion and submission of the survey. Respondents were informed of their right to ask questions or withdraw at any time. To comply with the General Data Protection Regulation (GDPR), all data were fully anonymized.

## 3 Results

### 3.1 Study population and characteristics

**Figure 3:**
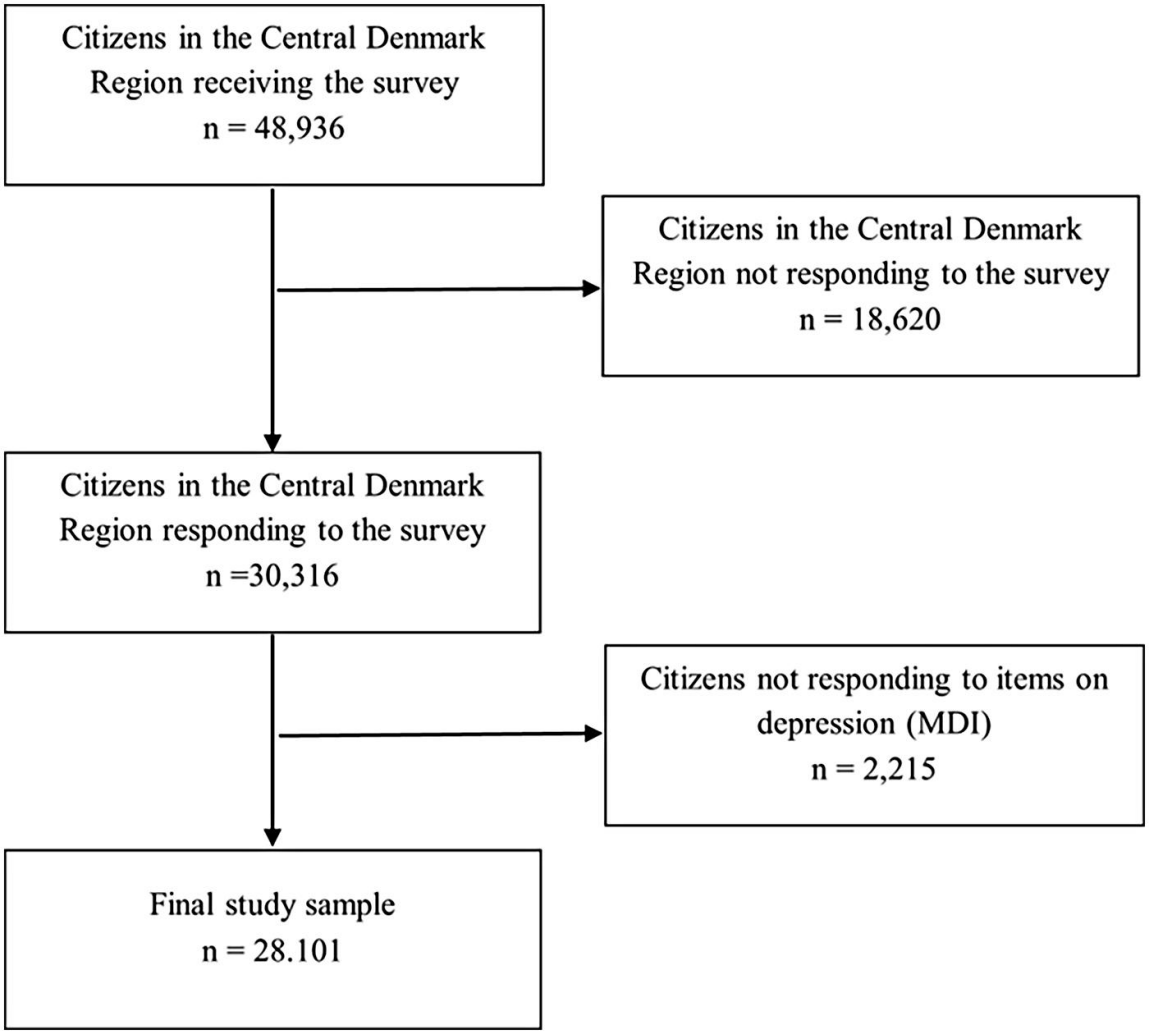
Flowchart of the study sample. Flowchart of the study sample showing participant selection, exclusions, and the final analytical sample included in the study.

A total of 48,936 received the survey, while 30,316 (62%) respondents completed it. Due to missing responses on the depression measure (MDI), the final analytical sample consisted of 28,101 (Figure 3).

Overall, 13% of the study sample reported having depression based on self-reported MDI scores (Table 2).

**Table 2:** Baseline characteristics of the study sample (*N* = 28,101).

Varibale	Depression *n* (%)	No depression *n* (%)
Total	3,060 (13.0)	25,041 (87.0)
*Body functions and body structures (34 items)*		
Sleep quality		
Poor sleep quality	1,331 (44.4)	1,973 (8.0)
Good sleep quality	1,599 (55.7)	22,074 (92.0)
Missing	130	994
Sleep duration		
Atypical sleep duration	1,829 (63.6)	8,124 (33.6)
Recommended sleep duration	1,077 (36.4)	16,569 (66.4)
Perceived stress		
Stress	2,621 (86.3)	3,923 (16.9)
No stress	429 (13.7)	20,960 (83.1)
Missing	10	158
Pain		
Pain	1,994 (63.6)	7,513 (29.5)
Less pain	1,059 (36.4)	17,453 (70.5)
Missing	7	75
Life strain		
Life strain	1,983 (67.9)	3,921 (17.2)
No life strain	1,001 (32.1)	20,745 (82.8)
Missing	76	375
Loneliness		
Lonely	1,382 (46.6)	1,581 (7.1)
Not lonely	1,657 (53.4)	23,292 (92.9)
Missing	21	168
BMI		
Overweight/obese	1,879 (61.3)	14,118 (56.4)
Underweight/normal weight	1,083 (38.7)	10,302 (43.6)
Missing	98	621
*Activities and participation (23 items)*		
Physical activity		
Physically inactive	956 (31.7)	4,013 (16.6)
Physically active	2,083 (68.3)	20,866 (83.5)
Missing	21	162
ADL		
Requires assistance with ADL	963 (30.7)	2,193 (8.8)
No assistance with ADL	2,050 (69.3)	22,542 (91.3)
Missing	47	306
Health literacy		
*Sub-scale: understanding health information:*		
Difficulty understanding health information	413 (13.0)	662 (3.0)
No difficulty understanding health information	2,611 (87.0)	23,954 (97.1)
Missing	36	425
*Subscale: engaging with healthcare providers:*		
Difficulty engaging with healthcare providers	776 (25.8)	1,093 (4.7)
No difficulty engaging with healthcare providers	2,251 (74.2)	23,529 (95.3)
Missing	33	419
Work and educational status		
Not engaged in work or education	1,483 (45.0)	10,263 (37.1)
Engaged in work or education	1,553 (55.0)	14,636 (62.9)
Missing	24	142
Work ability		
Reduced work ability	1,506 (47.8)	4,996 (19.3)
No reduced work ability	1,468 (52.2)	19,376 (80.7)
Missing	86	669
Social contact		
Reduced social contacts	1,273 (44.3)	5,097 (21.9)
Good social contacts	1,747 (55.7)	19,600 (78.1)
Missing	40	344
Community life		
Not involved in community life	1,734 (58.3)	8,547 (36.5)
Involved in community life	1,294 (41.7)	16,168 (63.5)
Missing	32	326
*Environmental factors (12 items)*		
Living with partner		
Living alone	1,196 (45.2)	5,715 (27.4)
Living with partner	1,819 (54.8)	19,100 (72.7)
Missing	45	226
Treatment recipient		
Yes	1,878 (57.9)	12,232 (45.6)
No	1,151 (42.1)	12,538 (54.4)
Missing	31	271
Income		
0–149,000	928 (34.5)	4,901 (20.3)
150,000–399,000	1,578 (50.8)	12,417 (50.4)
400,000+	438 (14.7)	6,787 (29.3)
Missing	116	936
Social security benefits		
Receiving social security benefits	1,232 (40.8)	3,030 (13.4)
Not receiving social security benefits	1,828 (59.2)	22,011 (86.6)
*Personal factors (11 items)*		
Age		
25–34	664 (30.6)	2,344 (15.4)
35–44	574 (20.2)	3,081 (15.3)
45–54	635 (19.4)	4,661 (19.3)
55–64	580 (15.2)	5,624 (18.9)
65–74	339 (7.8)	5,524 (17.4)
75+	268 (6.8)	3,807 (13.7)
Gender		
Male	1,160 (41.7)	11,659 (50,0)
Female	1,900 (58.3)	13,382 (50.0)
Educational attainment level		
No formal education or basic courses	901 (32.6)	5,031 (21.7)
Short-cycle tertiary education or vocational training	1,185 (37.0)	10,698 (42.1)
Medium- or long-cycle tertiary education	891 (30.4)	8,735 (36.2)
Missing	83	577
Native language		
Danish	2,641 (82.4)	23,461 (91.3)
Other	403 (17.6)	1,436 (8.7)
Missing	16	144
Smoking		
Smoker	792 (27.1)	3,513 (14.9)
Non-smoker	2,243 (72.9)	21,388 (85.1)
Missing	25	140
Alcohol		
Frequent heavy drinking	550 (25.5)	4,181 (20.8)
Infrequent or no heavy drinking	1,776 (74.5)	17,764 (79.2)
Missing	734	3,096

Frequencies (*n*) reflect the actual number of respondents, while percentages are weighted using survey weights to account for the sampling design. ADL, activities of daily living; BMI, body mass index.

Overall, respondents with self-reported depression showed profiles characterized by reduced functioning across all ICF components compared with those without depression (Table 2). As summarized in Table 2, they reported more impairments within body functions and body structures, more pronounced activity limitations and participation restrictions, and more constraining environmental and personal factors.

### 3.2 Associations between depression and functioning outcomes

In crude models, self-reported depression was strongly associated with a broad range of disability-related outcomes, including poor sleep quality, atypical sleep duration, perceived stress, pain, life strain, loneliness, overweight/obesity, physical inactivity, need for assistant with ADL, difficulty understanding health information, difficulty engagement with healthcare providers, lack of engagement in work or education, reduced work ability, reduced social contacts, and not involved in community life (Table 3).

**Table 3:** Associations between depression and functioning outcomes.

Outcomes	Crude OR (95% CI)	Adjusted Model 1 OR (95% CI)	Adjusted Model 2 OR (95% CI)
*Body functions and body structures*			
Poor sleep quality	9.20 (8.33; 10.18)	2.69 (2.30; 3.14)	2.94 (2.44; 3.54)
Atypical sleep duration	3.45 (3.15; 3.79)	1.32 (1.23; 1.41)	1.27 (1.18; 1.37)
Stress	31.13 (27.44; 35.32)	10.49 (8.94; 12.30)	9.75 (8.12; 11.72)
Pain	4.17 (3.80; 4.57)	1.20 (1.04; 1.38)	1.18 (0.99; 1.39)
Life strain	10.19 (9.25; 11.23)	2.53 (2.20; 2.90)	1.97 (1.67; 2.33)
Lonely	11.34 (10.24; 12.56)	3.12 (2.67; 3.66)	2.93 (2.43; 3.52)
Overweight/obese	1.23 (1.12; 1.35)	1.06 (0.93; 1.20)	1.14 (0.98; 1.32)
*Activities and participation*			
Physically inactive	2.34 (2.12; 2.58)	1.22 (1.05; 1.42)	1.12 (0.93; 1.34)
Requires assistance with ADL	4.61 (4.16; 5.11)	1.24 (1.03; 1.49)	1.39 (1.10; 1.74)
Difficulty understanding health information	4.91 (4.22; 5.71)	1.06 (0.79; 1.42)	1.17 (0.79; 1.72)
Difficulty engaging with healthcare providers	7.03 (6.25; 7.92)	1.99 (1.63; 2.43)	1.83 (1.44; 2.32)
Not engaged in work or education	1.39 (1.27; 1.52)	0.77 (0.67; 0.89)	1.40 (1.10; 1.79)
Reduced work ability	3.82 (3.48; 4.18)	1.12 (0.95; 1.33)	1.21 (0.96; 1.52)
Reduced social contacts	2.84 (2.59; 3.11)	1.18 (1.03; 1.36)	1.10 (0.94; 1.31)
Not involved in community life	2.43 (2.23; 2.66)	1.29 (1.14; 1.47)	1.19 (1.03; 1.39)

Estimates are based on survey-weighted logistic regression models. OR > 1 indicates increased odds of the specified outcome among individuals with depression compared with those without depression. Model 1: Adjusted for variables related to body functions and structure and to activities and participation. Model 2: Adjusted for variables in Model 1 and in addition to personal factors and environmental factors.

After adjustment for outcomes within the Body functions and structures and Activities and participation components (Model 1), most associations were substantially attenuated. Associations remained for poor sleep quality, atypical sleep duration, perceived stress, pain, life strain, loneliness, physical inactivity, need for assistant with ADL, difficulty engaging with healthcare providers, reduced social contacts, and lack of community participation (Table 3). Notably, respondents with self-reported depression were more likely to be engaged in work or education after adjustment. Further adjustment for personal and environmental factors (Model 2) yielded a similar pattern. Robust associations persisted between self-reported depression and poor sleep quality, atypical sleep duration, perceived stress, life strain, loneliness, need for assistance with ADL, difficulty engaging with healthcare providers, lack of engagement in work or education, not involved in community life. Changes in estimates across models reflect differences in adjustment for interrelated functioning domains and contextual factors. Notably, the variable “not engaged in work or education” changed direction, from 0.77 in Model 1 to 1.40 I Model 2, indicating a shift from a lower to a higher likelihood of the outcome after adjustment (Table 3).

## 4 Discussion

Overall, 13% of respondents reported symptoms consistent with depression, and these individuals exhibited substantially higher levels of impairment, activity limitation, and participation restriction across components of the ICF framework compared with those without depression. After adjustment for demographic, personal, and environmental factors, strong associations remained within impairment within body functions and body structures, particularly poor sleep quality, atypical sleep duration, perceived stress, life strain, and loneliness. Within activities and participation, self-reported depression was associated with activity limitations, including need for assistance with ADL, difficulties engaging with healthcare providers, and lack of engagement in work or education, and restricted participation in community life. These findings indicate that the associations with self-reported depression are particularly pronounced in domains related to daily coping, social relations, and participation in everyday roles. The attenuation of associations from crude to adjusted estimates suggests that limitations in functioning tend to cluster across domains. However, as adjusted models included variables that may lie on the pathway between depression and functioning, some degree of overadjustment cannot be excluded, and these estimates should be interpreted with caution. While additional adjustment for personal and environmental factors did not substantially alter the overall pattern of association, residual confounding and potential bidirectional relationships cannot be excluded. Associations that remained strong after adjustment may therefore reflect domains most closely linked to depression. From an ICF perspective, these findings highlight the interconnected nature of functioning domains rather than distinct causal pathways. Accordingly, the results should be interpreted as exploratory associations rather than estimates of total effects.

Our results are consistent with current evidence showing that depression is associated with disability across physical, cognitive, social, and occupational domains (4, 5, 7, 8, 15, 46–48). Disability often persists after symptom improvement (15), and variation in functioning cannot be explained solely by symptom severity. Personal and environmental factors, including coping, workplace conditions, social support, and quality of life, account for substantial additional variance (4, 5, 47). The persistent associations we observed for stress, sleep problems, life strain, loneliness, ADL difficulties, and reduced participation are consistent with previous research and highlight the multidimensional nature of depression-related disability. Taken together, this literature supports the importance of functioning outcomes as essential endpoints in clinical management and rehabilitation for depression, rather than relying on symptom reduction alone (11).

In this study depression was measured using self-reported MDI, a validated instrument suitable for population-based research. The MDI has demonstrated good clinical validity, showing strong correlations with clinical-rated measures of depression severity and acceptable agreement with diagnostic criteria (21), although it does not provide a formal clinical diagnosis and may introduce reporting and social desirability bias. The use of sum scores enables capture of a broader spectrum of symptom severity compared to algorithm-based classifications, which may underestimate depressive symptoms in population samples (49). Sum scores are therefore considered more sensitive in population-based research (49, 50). This approach is particularly relevant as individuals with mild or moderate symptoms, who may not meet diagnostic criteria, also experience substantial limitations in functioning. Much existing research has focused on patients with major depressive disorder, where pronounced cognitive, social, and occupational impairments and persistent disability are well documented (3–5, 8, 12, 15). Our findings extend this evidence by demonstrating that disability is present across the full spectrum of symptom severity. Depression was analyzed as a dichotomous variable. Although categorization into severity levels could have enabled exploration of dose–response relationships, this was not feasible due to the relatively low prevalence of depression in the sample (13%), which would have resulted in small subgroup sizes and limited statistical power.

The ICF framework guided the interpretation of associations between self-reported depression and functioning. Its biopsychosocial structure helped situate findings across body functions and body structures, activities and participation, and contextual factors, reflecting the multidimensional nature of health and disability (13). Applying the refined ICF Linking Rules (22) to a population survey such as “How are you?” was informative but highlighted practical challenges. As the survey was designed to measure general health and well-being rather than functioning, several items spanned more than one ICF category or combined elements from different components (18). This illustrates a broader methodological issue when linking secondary data to ICF. Population surveys often lack the granularity needed to map cleanly onto the classification's conceptual distinctions (22, 51). Nonetheless, the linking process enabled transparent categorization of items and allowed identification of consistent patterns of disability across all ICF components. Using ICF in a population-level analysis raises important interpretive considerations. The framework is designed to capture functioning at the individual level, whereas our analyses describe aggregate patterns. This inevitably smooths over individual variability that is central to ICF-based assessments in clinical practice (52). Furthermore, linking inherently involves interpretation, particularly when items are broad, multidimensional, or only indirectly related to functioning. Rather than undermining the framework, this highlights the importance of transparency when applying ICF Linking Rules and of acknowledging the conceptual distance between administrative survey items and ICF categories. Despite these constraints, the use of ICF offers clear advantages for rehabilitation research. It provides a coherent structure for integrating cognitive, emotional, social, and environmental aspects of functioning, thereby helping to identify needs that may be overlooked when care remains focused on symptomatology. The classification also supports communication across sectors, by offering a shared language for describing functioning and disability (14, 53). In settings where depression contributes to reduced work ability, challenges with self-management, or barriers to participation, such common terminology may facilitate more coordinated and person-centred rehabilitation planning (4). Overall, the findings demonstrate the value of applying an ICF perspective in population-based mental health research. Even when working with secondary data, the framework helps to contextualize depressive symptoms within the broader lived experience of functioning and thereby strengthens the relevance of research for rehabilitation practice and policy.

This study has several methodological strengths. It draws on data from a large population-based sample of more than 28,000 adults, which supports representativeness and enables robust analyses across multiple ICF domains. The relatively high response rate of 62%, together with weighting for sampling design and non-response, supports the generalizability of the findings (44). In addition, the use of validated instruments strengthens measurement validity and reliability. The systematic application of ICF Linking Rules (22) and cross-checking with the ICF Core Set for Depression (14) further supports the conceptual rigor of the study, however we acknowledge that some degree of subjectivity cannot be fully eliminated. Several limitations should also be acknowledged. Due to the cross-sectional design we cannot draw conclusions about causal relationships between self-reported depression and disability. Another limitation is that the study relies on self-reported data, which may introduce information bias including recall bias and social desirability bias. Respondents may underreport or overreport symptoms of depression and aspects of functioning, which could lead to misclassification and influence the observed associations. However, most variables were assessed using validated instruments, which strengthens data quality and reduces the likelihood that these limitations have materially influenced the overall pattern of associations. The survey items were not originally developed to capture functioning within the ICF framework, and specific measures and cut-offs, although informed by prior research, have not been formally validated for this population. Selection bias may have occurred due to both item-level missingness and survey non-response. Individuals with poor mental health may have been less likely to participate in or complete the survey, potentially resulting in their underrepresentation in the study sample. Consequently, the associations between depression and functioning may be underestimated.

Although calibrated non-response weights were applied to improve representativeness (54), such methods cannot fully eliminate this potential source of bias. We did not formally assess the missing data mechanism, and missingness may therefore not be random. Data were collected during the COVID-19 pandemic, a period characterized by social restrictions and changes in daily life, which may have influenced levels of stress, loneliness, sleep, and changes in daily routines (55). These conditions may have amplified symptom reporting, potentially leading to an overestimation of the observed associations. Accordingly, caution is warranted when generalizing the findings to non-pandemic contexts. Differences between countries in welfare structures, access to health and rehabilitation services, and cultural understanding of depression and functioning may influence how self-reported health are experienced and reported (56). These contextual variations may influence the extent to which the findings can be directly applied to other countries.

The findings highlight the importance of addressing functioning across multiple domains in rehabilitation for individuals with depression. Although symptom severity is an important indicator of need, our results show that many individuals report marked difficulties in central domains such as life strain, ADL and participation, even at lower levels of symptom severity. This suggests that symptom-based screening alone may overlook individuals with substantial disabilities. Incorporating routine assessment of functioning in both clinical and community settings could support earlier identification of these difficulties and facilitate more targeted rehabilitation (4). The pattern we observe, with prominent problems in life strain, everyday activities and social participation, indicates where rehabilitation efforts may need to be prioritized. These findings highlight the importance of coordinated, cross-sectoral collaboration between mental health services, social services, and employment sectors to support trajectories and reduce the risk of long-term disability; a conclusion that is also supported by previous research (57).

Future research should use longitudinal designs with repeated measures of both depressive symptoms and functioning across key ICF domains to clarify temporal and potential bidirectional relationships. For example, prospective cohort studies with multiple follow-up point could examine whether changes in sleep, stress, ADL performance, and social participation precede or follow changes in symptom severity. In addition, studies should incorporate clinical validation of depression, such as structured diagnostic interviews, alongside self-reported measures to reduce misclassification. Linking survey data with registry-based information would further allow identification of trajectories of functioning and of subgroups at risk of persistent disability. Such approaches would strengthen the evidence base for ICF-informed approaches in rehabilitation and support the development of more targeted and timely interventions.

## 5 Conclusions

This large population-based study demonstrates that self-reported depression is associated with impairments, activity limitations and participation restrictions across ICF components. Even after adjustment for personal and environmental factors, strong associations remained for sleep quality and duration, perceived stress, life strain, loneliness, need for assistance with ADL, difficulties engaging with healthcare providers, and reduced participation in work, education, and community life. Together these findings highlight the broad distribution of depression on functioning and support the relevance of the ICF framework for understanding functioning and disability in mental health. Future longitudinal studies are needed to clarify temporal relationships and further explore the role of ICF-based approaches in rehabilitation.

## Data Availability

The raw data supporting the conclusions of this article will be made available by the authors, without undue reservation.
